# Rare variation in neurological disease genes and its role in multiple sclerosis mimicry and phenotype

**DOI:** 10.1186/s13073-025-01582-x

**Published:** 2025-12-10

**Authors:** Nicholas B. Blackburn, Bennet J. McComish, Allan Motyer, James C. Slimmer, Stephen J. Leslie, Simon A. Broadley, Vilija G. Jokubaitis, Anneke Van Der Walt, Allan G. Kermode, Jeannette Lechner-Scott, Grant P. Parnell, Marzena J. Fabis-Pedrini, Rodney J. Scott, Stacey Jackson, Vicki E. Maltby, Jac C. Charlesworth, Kathryn P. Burdon, Bruce V. Taylor, Trevor J. Kilpatrick, Justin P. Rubio

**Affiliations:** 1 Menzies Institute for Medical Research, University of Tasmania, Hobart, TAS 7000 Australia; 2 School of Mathematics and Statistics, The University of Melbourne, Parkville, VIC 3052 Australia; 3 Melbourne Integrative Genomics, The University of Melbourne, Parkville, VIC 3052 Australia; 4 Menzies Health Institute Queensland, Griffith University, Gold Coast, QLD 4215 Australia; 5 Department of Neuroscience, Monash University, Melbourne, VIC 3004 Australia; 6 Department of Neurology, Alfred Health, Melbourne, VIC 3004 Australia; 7 Perron Institute for Neurological and Translational Science, QE II Medical Centre, The University of Western Australia, Perth, 6009 Australia; 8 Personalised Medicine Centre, Health Futures Institute, Murdoch University, Perth, 6150 Australia; 9 Department of Neurology, John Hunter Hospital, Newcastle, NSW 2305 Australia; 10 School of Medicine and Public Health, The University of Newcastle, Newcastle, NSW 2308 Australia; 11 Hunter Medical Research Institute, The University of Newcastle, Callaghan, NSW 2305 Australia; 12 Centre for Immunology and Allergy Research, Westmead Institute for Medical Research, University of Sydney, Sydney, NSW 2145 Australia; 13 School of Medical Sciences, Faculty of Medicine and Health, The University of Sydney, Sydney, NSW 2050 Australia; 14 Division of Molecular Medicine, NSW Health Pathology-North, John Hunter Hospital, New Lambton Heights, NSW 2305 Australia; 15 The Florey Institute of Neuroscience and Mental Health, Parkville, VIC 3052 Australia; 16 Florey Department, The University of Melbourne, Melbourne, VIC 3010 Australia; 17 Department of Neurology, Royal Melbourne Hospital, Melbourne, VIC 3052 Australia

**Keywords:** Multiple sclerosis, Genetic factors, Exome sequencing, Misdiagnosis, Multimorbidity

## Abstract

**Background:**

Multiple sclerosis (MS) diagnosis relies on identifying disease episodes disseminated in space and time, and excluding other disease explanations. MS is a genetically complex autoimmune and neurodegenerative disorder that shares features with some monogenic progressive neurological conditions. The extent to which people diagnosed with MS have an alternate diagnosis (MS mimic), or genetic multimorbidity is unknown. Additionally, the burden of rare variation associated with MS risk and severity in monogenic neurological disease genes has not been evaluated. We investigated the prevalence of disease-causing variants in progressive neurological disease genes, and their contribution to MS risk and severity, in 4,340 MS cases diagnosed in sub-speciality clinics in Australia and New Zealand, and in 2,861 local controls.

**Methods:**

Exome sequencing and array-based genotyping data were analysed for 1,680 genes with pathogenic or likely pathogenic variants reported in ClinVar. Clinical history reviews of MS cases with putative disease-causing variants were conducted. We specifically examined the contribution of rare, likely deleterious variants in a subset of 30 hereditary spastic paraplegia (HSP) genes in 421 individuals with progressive onset MS (POMS). Gene-based association tests with MS risk and severity were performed for all genes in the cohort.

**Results:**

We identified 166 MS cases (3.82%) with variants prompting clinical history reviews, and of 75 cases reviewed, four (0.13% of all cases) had either genetic multimorbidity in addition to MS or a potential misdiagnosis. In contrast to previous findings we observed no enrichment of likely deleterious variants in HSP genes in POMS, nor did we find significant associations between neurological disease genes and MS risk or severity.

**Conclusions:**

Our findings suggest that rare deleterious genetic variation in progressive neurological disease genes does not play a substantive role in MS risk or severity, and that misdiagnosis is exceedingly rare in this cohort. Consequently, among individuals diagnosed with MS by a specialist, a very small proportion may benefit from clinical genomic testing to inform MS diagnosis or an alternate diagnosis, which could have implications for healthcare management.

**Supplementary Information:**

The online version contains supplementary material available at 10.1186/s13073-025-01582-x.

## Background

 Multiple sclerosis (MS) is a complex autoimmune and neurodegenerative disease [1]. The 2017 McDonald criteria used to diagnose MS emphasises that a diagnosis is only made when “there is no better explanation” [2]. Disease complexity and the criteria used for diagnosis contribute to a frequency of misdiagnoses of MS ranging between 5%−10%, even when the McDonald criteria are fulfilled [3]. The implications of an MS misdiagnosis can have significant clinical, personal and economic ramifications, including that a person may be exposed unnecessarily to side effects associated with disease-modifying therapy (DMT) [4].

The clinical spectrum of MS, which classifies individuals based on disease course into a relapsing onset MS (ROMS) or a progressing onset MS (POMS), shares features with monogenic neurological conditions including “MS mimic” diseases [5, 6]. These “MS mimic” diseases can have paraclinical features similar to those that are used during a differential diagnosis of MS, such as the presence of white matter lesions on magnetic resonance imaging (MRI) scans [7]. Most monogenic neurological conditions do not overlap with MS with respect to MRI features but some overlap in physical symptoms and might co-occur with MS as a multimorbidity. Genetic diseases sometimes misdiagnosed as MS include Fabry disease [8], cerebral autosomal dominant arteriopathy and subcortical infarct leukoencephalopathy (CADASIL) [9], leukodystrophy [10] and hereditary spastic paraplegia (HSP) [11]. The progressive leg weakness that occurs in HSP, for example, is one of the most common symptoms of POMS, however, genetic testing for HSP and other genetically determined MS mimics is not currently a routine component of MS diagnosis [2] and therefore, the true frequency of misdiagnosis, or multimorbidity with a monogenic disease, is unknown.

Previously, Jia et al. (2018) [12] presented results from a genome sequenced discovery cohort of 38 people diagnosed with POMS and 81 controls with variants of interest genotyped in an additional 746 POMS cases, 3,049 ROMS cases and 1,000 controls. In their study, Jia et al. discovered individuals with potentially pathogenic mutations in clinically implicated genes for spastic paraplegias and megalencephalic leukodystrophy. These findings aligned with reports of the co-occurrence of MS and spastic paraplegias in the same individual [13], and the results of a recent systematic review that identified 20 possible co-occurrences [14]. Recently, Mandler et al. (2024) [15], searched for MS mimics in a cohort of 278 patients diagnosed with MS using exome sequencing, including 18 individuals with POMS. They applied a curated gene list of 495 monogenic disease genes for diseases that share features with MS and identified in their cohort a single definite MS misdiagnosis with the individual carrying an established CADASIL causing *NOTCH3* variant [15]. Both of the aforementioned studies approached the identification of MS mimics in their cohorts using restricted or customised gene lists. Broader, neurological disease-orientated gene panels, such as PanelApp Australia’s neurology and neurodevelopmental disorder gene panels [16], have not been investigated in MS. Such panels are the result of collective community efforts to curate disease gene panels, and by drawing on these panels potential investigator bias on gene selection can be reduced. Further, expanding the spectrum of progressive neurological disease genes considered in MS is warranted given findings that suggest biological connections between the genetic architecture of MS to monogenic disorders [17].

When an individual with an MS diagnosis receives a genetic mimic diagnosis, the challenge is whether the individual has both MS *and* the mimic disease (multimorbidity), or only the mimic disease [13]. If the individual has both diseases, there may be difficulty in establishing which clinical features belong to which disease and whether the mimic disease is modifying the course of MS. Further, the hypothesis that an accumulation of potentially damaging genetic variation in MS mimic genes may influence MS risk or modify disease outcome has been proposed previously [12]. However, this hypothesis has not been comprehensively explored in MS via genomic sequencing of genes for progressive monogenic neurological diseases, which collectively encompass potential MS mimic genes.

To address this gap, we have undertaken exome sequencing on a large sample of MS cases and population controls collected by the Australia and New Zealand MS Genetics Consortium (ANZgene). We hypothesised that for some individuals diagnosed with MS there are variants of established clinical relevance that explain some, or potentially all, of their neurological symptoms. We also posited that a burden of generally rare, likely deleterious variations in a subset of monogenic progressive neurological disease genes are associated with MS disease risk and/or disease severity.

## Methods

### Study participants

MS cases were recruited via convenience sampling at outpatient MS subspecialty clinics at multiple centres across New Zealand and four states of Australia from 1998-2022. Recruitment sites included the Alfred Hospital, and Royal Melbourne Hospital (Melbourne, Victoria), John Hunter Hospital (Newcastle, New South Wales), Sir Charles Gairdner Hospital (Perth, Western Australia), Royal Hobart Hospital (Hobart, Tasmania). All cases recruited at these sites met the 2017 McDonald diagnostic criteria for MS [2]. A subset of cases (*n* = 941) recruited at the Royal Melbourne Hospital had either definite MS, clinically definite MS or laboratory-supported definite MS according to the 2001 McDonald and Poser MS diagnostic criteria, respectively [18, 19]. MS cases from New Zealand were recruited as part of the New Zealand MS prevalence study [20] and met the 2005 McDonald diagnostic criteria for MS [21]. Together these cases comprise ANZgene; a multi-centre Australia and New Zealand MS Genetics Consortium (Additional file 1: Method S1). Population controls were recruited through the Australian Bone Marrow Donor Registry (ABMDR), and unaffected controls from within ANZgene cohorts. Additional file 2: table S1 details the cohort characteristics corresponding to the participants for whom data was generated in this study.

### DNA samples and genetic data

Genomic DNA (gDNA) from Australian cases and controls was extracted from whole blood using a variety of standard laboratory approaches. gDNA from New Zealand MS cases, and nine Tasmanian cases, was isolated from saliva self-collected into Oragene DNA tubes according to the manufacturer’s instructions (DNA Genotek). DNA was aliquoted into 384-well plates and sent to the Regeneron Genetics Center (RGC, Tarrytown, NY, USA) for further quality control prior to library preparations for exome sequencing and array-based genotyping. Genetic data underwent rigorous quality control (Additional file 1: Method S1), generating the final exome sequencing and array-based genotype datasets used in this study (Additional file 1: Fig S1).

### Identifying clinically implicated variants in progressive neurological disease genes in MS cases

To identify variants in established progressive neurological disease genes, we drew on an amalgamated collection of neurological and neurodevelopmental disorder clinical genomics gene panels from PanelApp Australia [16] – the Progressive Neurological Diseases Superpanel ‘Green’ tier gene list (v14.477). Corresponding to the limits of the exome sequencing data in this project, we restricted our analysis to a subset of this Superpanel encompassing 1,680 nuclear genes for which single nucleotide variants (SNVs) or small insertion/deletion variants (indels) cause disease. Variants identified in the exome sequencing data were annotated using Ensembl’s Variant Effect Predictor (VEP, v110) [22], and analysis restricted to variants annotated to the 1,680 genes in the Superpanel. Variant annotation files generated were processed in R (v4.2.0, http://R-project.org) using the tidyverse suite of packages [23]. Variant annotations were processed in these genes to identify variants with annotations of ‘Likely Pathogenic’ and/or ‘Pathogenic’ variants recorded in ClinVar [24], using this database as a source of evidence for clinically described disease-causing variants, with analysis restricted to MS cases (detailed further in Additional file 1: Method S2) to prioritise individuals for blinded clinical history reviews. This approach applies the reported variant-phenotype associations documented in ClinVar to search for corresponding evidence in the clinical histories of MS cases.

### Blinded clinical history reviews

A blinded review process was designed to facilitate an unbiased clinical history review of prioritised variants in the corresponding MS cases. Each case prioritised with a clinically implicated variant was referred to the contributing ANZgene MS clinician (A.V.D.W., A.G.K., J.L-S., B.V.T., T.J.K.) without revealing the genetic finding. Clinicians, where possible, were asked to review the available clinical history of the individual and extract relevant information, including the individual’s family history, disease course and diagnosis, and any features of their history that could be considered inconsistent with an MS diagnosis. Following completion of the reviews, a follow-up meeting was held with each clinician to unblind the genetic result and discuss whether there was evidence of a plausible phenotypic effect in the context of the genetic finding. A consensus was then reached on a case-by-case basis as to whether the variant had evidence of an effect, whether the variant was influencing the MS clinical course, and whether the individual potentially had a multimorbidity, or an MS mimic disease.

### Statistical analysis

#### Comparison of variants in HSP genes in POMS cases and controls

Previously, Jia et al. investigated whether people with POMS have a higher number of rare, coding and splicing variants in 30 genes known to cause HSPs [12]. We replicated their analysis in the current dataset using the same set of 169 variants (Additional file 2: table S4). The total number of qualifying variants was calculated per person. As per Jia et al., logistic regression, adjusting for sex, was used to model the probability of POMS as a function of the total number of variants per person. We additionally performed a Firth penalized logistic regression to account for event rarity in the analysis, and compared the mean number of variants in each group using a *t*-test. Statistical tests were performed in R (v4.2.0, http://R-project.org).

### Gene-based burden testing of progressive neurological disease genes in cases and controls

To test for an association between a burden of variation in progressive neurological disease genes and MS, we conducted gene-based association testing (Additional file 1: Method S3). All cases were analysed together as one MS group, and separately as ROMS and POMS groups. We also tested for gene-based associations with MS disability and disease progression using the Age Related Multiple Sclerosis Severity Score (ARMSS), which was calculated from cross-sectional Expanded Disability Status Scale (EDSS) scores as per Manouchehrinia et al. [25], and analysed separately for ROMS and POMS individuals. Gene-based testing was performed using the Sequence Kernel Association Test – Optimal (SKATO) [26], implemented in regenie (v3.4.1) and implementing the Cauchy combination method modification (SKATO-ACAT) for combining *P*-values from SKATO models [27]. FDR adjusted *P*-values were calculated within each group analysis.

## Results

A total of 8,266 samples were exome sequenced in this study (Additional file 1: Fig. S1). After quality control, data was available for 4,340 MS cases and 2,861 controls for the MS mimic analysis (7,201 exomes total). For gene-based association testing, matched exome and Illumina Global Screening Array (GSA) SNP genotyping data was available for 3,172 MS cases and 2,637 controls. Table 1 and Table S1 summarise the overall cohort characteristics.

**Table 1:** Demographic, clinical, and data characteristics of the anzgene WES-GSA cohort

		Cohort by clinical characteristics		
		ROMS^a^	POMS^b^	Population Controls
Characteristics		3,978	497	2,962
Sex, n (%)	Female	3,089 (77.7)	294 (59.2)	1,771 (59.8)
	Male	889 (22.3)	203 (40.8)	1,191 (40.2)
Age at first symptom	Median, (IQR^d^) NA^e^	32.0 (26.0, 40.0) 116	42.0 (34.9, 48.9) 2	--
Disease duration, years	Median, (IQR^d^) NA^e^	13.0 (6.3, 21.1) 119	15.0 (8.0, 23.0) 4	--
ARMSS^c^ score	Median, (IQR^d^) NA^e^	5.0 (2.5, 7.4) 123	5.1 (2.7, 7.2) 3	--
WES^f^ & GSA^g^		2,751	421	2,637
WES^f^ only		1,105	63	224
GSA^g^ only		122	13	101

^a^ROMS = Relapsing onset MS, including 2,717 relapsing-remitting MS, 1,099 secondary progressive MS, 53 clinically isolated syndrome, 109 MS subtype unspecified, ^b^POMS = Progressive onset MS, including 480 primary progressive MS, 17 progressive relapsing MS, ^c^ARMSS = age-related multiple sclerosis severity, ^d^IQR = interquartile range, ^e^NA = missing data, ^f^WES = Whole Exome Sequencing, ^g^GSA = Illumina Infinium Global Screening Array

### Identification of likely pathogenic and pathogenic variants in progressive neurological disease genes

**Fig. 1:**
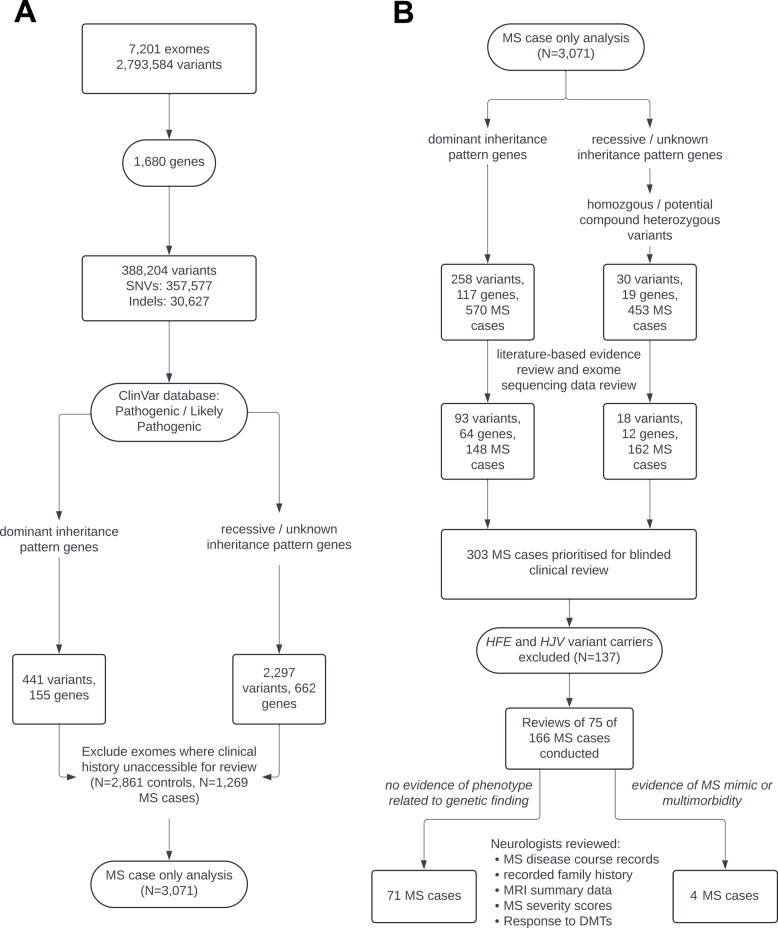
Flow chart depicting approaches used to identify variant carriers. **A** Identification of likely pathogenic/pathogenic variant carriers in exome sequencing data. **B** Prioritisation of MS cases for clinical history reviews

Figure 1A illustrates the process of identifying likely pathogenic and pathogenic variants within the exome sequence data. With follow-up analysis restricted to MS cases only, Fig. 1B illustrates the process undertaken to prioritise 303 MS cases for blinded clinical history reviews. Review was not possible for all prioritised individuals due to a lack of available clinical information. As individuals with variants in hemochromatosis genes, *HFE* or *HJV*, (and no other gene) accounted for 137 individuals (45.5%) these were deprioritised. We note that the high number of hemochromatosis variant carriers in this cohort can be attributed to linkage disequilibrium between *HFE* Cys282Tyr and the HLA-DRB1*15:01 MS risk allele [28]. For the remaining 166 MS cases (Additional file 2: Table S5), 75 (45.2%) clinical history reviews were conducted. As relatives were excluded during the exome sequencing data quality control process, we confirmed that none of the 166 MS cases prioritised for clinical history review were a relative of an excluded sample. Clinical records for four of 75 MS cases (5.3%) showed clear evidence of a phenotype that could be attributed to the genetic finding. Alternatively, it was considered that the lack of attributable variant to disease association for the other 71 cases may either be due to inaccurate variant classifications in the ClinVar database [29], variable penetrance, the implicated disease being a late-onset condition, or variable expressivity, where the variant effect may be masked by MS.

Clinical information for the four cases with genetic evidence of either an MS mimic disease or a multimorbidity are shown in Table 2. Detailed clinical histories for these individuals are described below.

**Table 2:** Four individuals with MS with genetic mimicry or Multimorbidity

ID	Geno-type	Variant Identifiers	Variant Consequence	Gene Symbol	ClinVar Variation ID	ClinVar Classification	ClinVar Clinical Conditions	Sex	MS	Age at onset (first symptom)	Age at diagnosis	Age at assessment	Disease duration at assessment (years)	EDSS
94	1/1	11:64759751:G:A (p.Arg50Ter)	stop_gained	*PYGM*	2298	Pathogenic	Glycogen storage disease, type V	F	ROMS	20–29	20–29	40–49	20	4
108	0/1	12:57569263:A:G (p.Tyr276Cys)	missense_variant	*KIF5A*	6808	Pathogenic/Likely Pathogenic	Hereditary spastic paraplegia 10	F	POMS	30–39	30–39	30–39	7	4
131	0/1	19:15185593:G:A (p.Arg680Cys)	missense_variant	*NOTCH3*	1,676,246	Conflicting	Cerebral arteriopathy, autosomal dominant, with subcortical infarcts and leukoencephalopathy, type 1	M	ROMS	40–49	NA	50–59	6	1.5
164	0/1	19:15192218:G:A (p.Arg141Cys)	missense_variant	*NOTCH3*	447,846	Pathogenic	Cerebral arteriopathy, autosomal dominant, with subcortical infarcts and leukoencephalopathy, type 1	M	ROMS	< 20	NA	50–59	46	6.5

### Clinical history summaries of identified MS mimic or multimorbidity cases

#### *KIF5A* (NM_004984.4):c.827 A > G (p.Tyr276Cys)

An established pathogenic variant for HSP in *KIF5A* [30] was found in a female with POMS, fulfilling the McDonald criteria 2017, symptom onset at age 30–39. There was a family history of Charcot-Marie Tooth disease and spastic paraplegia. Her clinical features included neuropathy consistent with HSP. Examination of MRI was consistent with an MS diagnosis. Specifically, MRI scans revealed multiple T2 hyperintensities in the periventricular subcortical, posterior fossa and spinal cord regions (spinal cord lesions were discrete ovoid eccentric lesions). Many periventricular lesions were perpendicular to the body of the lateral ventricles and/or callosal junction (Dawson’s Fingers). Oligoclonal band status was not assessed. It was concluded that this patient had both POMS, and HSP.

#### *NOTCH3* (NM_000435.3):c.2038 C > T (p.Arg680Cys)

A male diagnosed with ROMS at age 40–49 was identified carrying an established pathogenic variant for CADASIL in *NOTCH3* [31]. The diagnosis of ROMS was made six years after the onset of neurological symptoms and the available clinical records pertain to a single time-point when the participant was recruited to an MS genetic research study. The individual presented with headache, diplopia and subsequently with balance issues. Ongoing vision and balance issues were recorded and the individual described memory loss, although there was no evidence of progressive dementia at the time of recruitment. Oligoclonal band status was not assessed. Review of the MRI history showed that radiology interpretations had varied with the suggestion that MRI features were more consistent with a vascular disease rather than a demyelinating disease. MRI showed Fluid-Attenuated Inversion Recovery (FLAIR) lesions consistent with chronic infarcts, however no lesions in typical CADASIL regions were identified. There was no family history of neurological illness, including CADASIL, volunteered. It was concluded that this patient had a potential misdiagnosis of MS. The 19:15185593:G: A variant was reported as ‘likely pathogenic’ in the earlier database version of ClinVar v20220910, leading to the review reported here, but is currently classified as ‘conflicting interpretations of pathogenicity’ in v20240107.

#### *NOTCH3* (NM_000435.3):c.421 C > T (p.Arg141Cys)

A male originally diagnosed with ROMS at under 20 years of age was identified carrying an established pathogenic variant for CADASIL in *NOTCH3* [31]. The clinical records reviewed for this participant pertain to a single time point at recruitment to an MS genetic research study. Upon re-review of the clinical records for the current study the individual was revealed to have an MRI that was atypical for a diagnosis of MS. The individual’s clinical presentation was with a left hemiparesis and incoordination. The brain MRI revealed widespread cerebral atrophy and confluent areas of T2 hyperintensity with clear-cut involvement of the temporal lobes and cystic transformation involving the thalamus, basal ganglia and corona radiata. Oligoclonal band status was not assessed. There was a five year history of emergent memory problems, commencing four years after the onset of neurological symptoms (Kurtzke Functional Score = 2). There was no family history of neurological illness, including CADASIL volunteered. It was concluded that this patient had a potential misdiagnosis of MS.

#### *PYGM* (NM_005609.4):c.148 C > T (p.Arg50Ter)

A female diagnosed with ROMS at 20–29 years of age was shown to be homozygous for a pathogenic variant in *PYGM*, an established cause of McArdle disease [32]. Clinical history review revealed that McArdle disease was already established, consistent with the genetic finding and that otherwise, this individual met the 2017 McDonald criteria for an MS diagnosis. It was concluded that this patient had both MS and the multimorbidity of McArdle disease.

### Gene-based analysis of rare deleterious variant enrichment in progressive neurological disease genes

#### HSPgenes

**Fig. 2:**
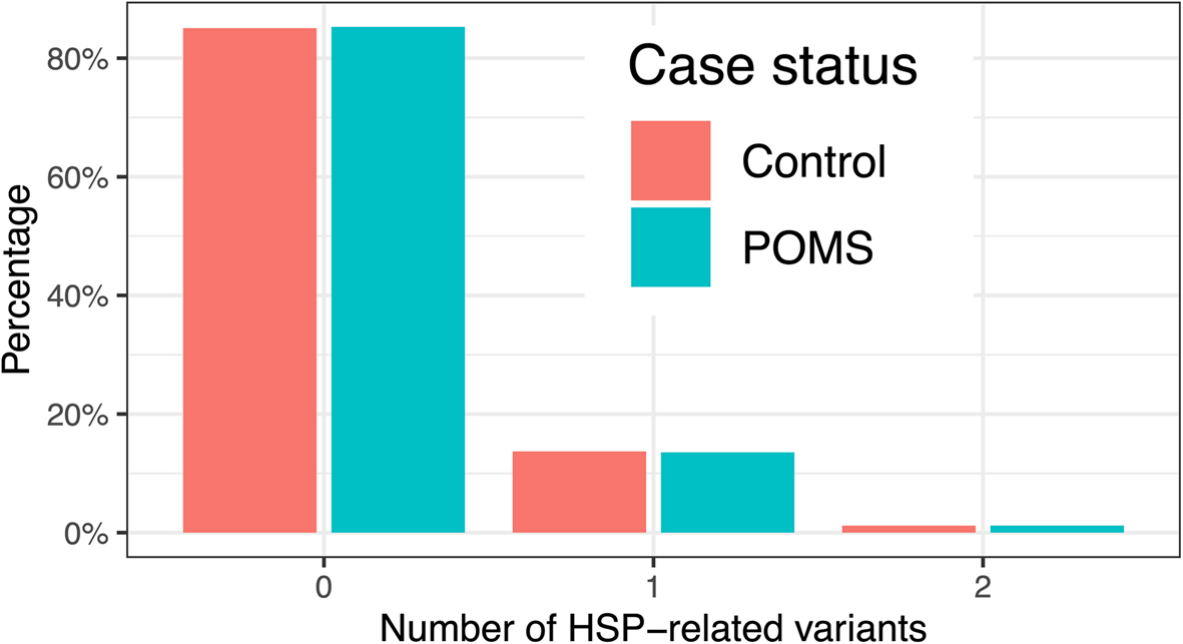
Frequency of 169 variants in 30 HSP genes in progressive onset MS (POMS) cases and controls. Bar plots showing the frequency of POMS cases and controls that have variants in HSP genes. There was no difference identified between groups in this cohort

Previously Jia et al. [12] identified that POMS (primary progressive) MS cases had, on average, more variants in HSP genes than controls. Specifically, they tested for 169 variants across 30 genes that were present on the MS replication SNP array. For POMS, across both their discovery (48 cases and 100 controls) and replication (266 cases and 887 controls) cohorts, 51 variants were detected. To replicate the previous findings from Jia et al. [12], we used exome sequencing data from 421 POMS cases and 2,637 population controls, to investigate the same set of 169 variants in HSP genes (Additional file 2: Table S4). In total, 57 of the 169 variants were detected in either POMS cases or controls in our cohort. In line with Jia et al. [12], we used logistic regression to compare the number of HSP variants as a continuous predictor between groups. As there were two control individuals that each had three variants, we collapsed these into the two variant category for analysis. In contrast to previous findings, we found no difference between groups (Fig. 2) using a standard logistic regression (β = − 0.018, 95% CI: [−0.28, 0.24], *P* = 0.89), nor a Firth penalized logistic regression (β = − 0.012, 95% CI: [−0.28, 0.24], *P* = 0.93). Additionally, the average number of variants did not differ between POMS cases (mean = 0.16) and controls (mean = 0.16), as confirmed using a Welch’s *t*-test (t(565.24) = 0.13, *p* = 0.89, 95% CI [–0.038, 0.044]).

### Other progressive neurological disease genes

**Fig. 3:**
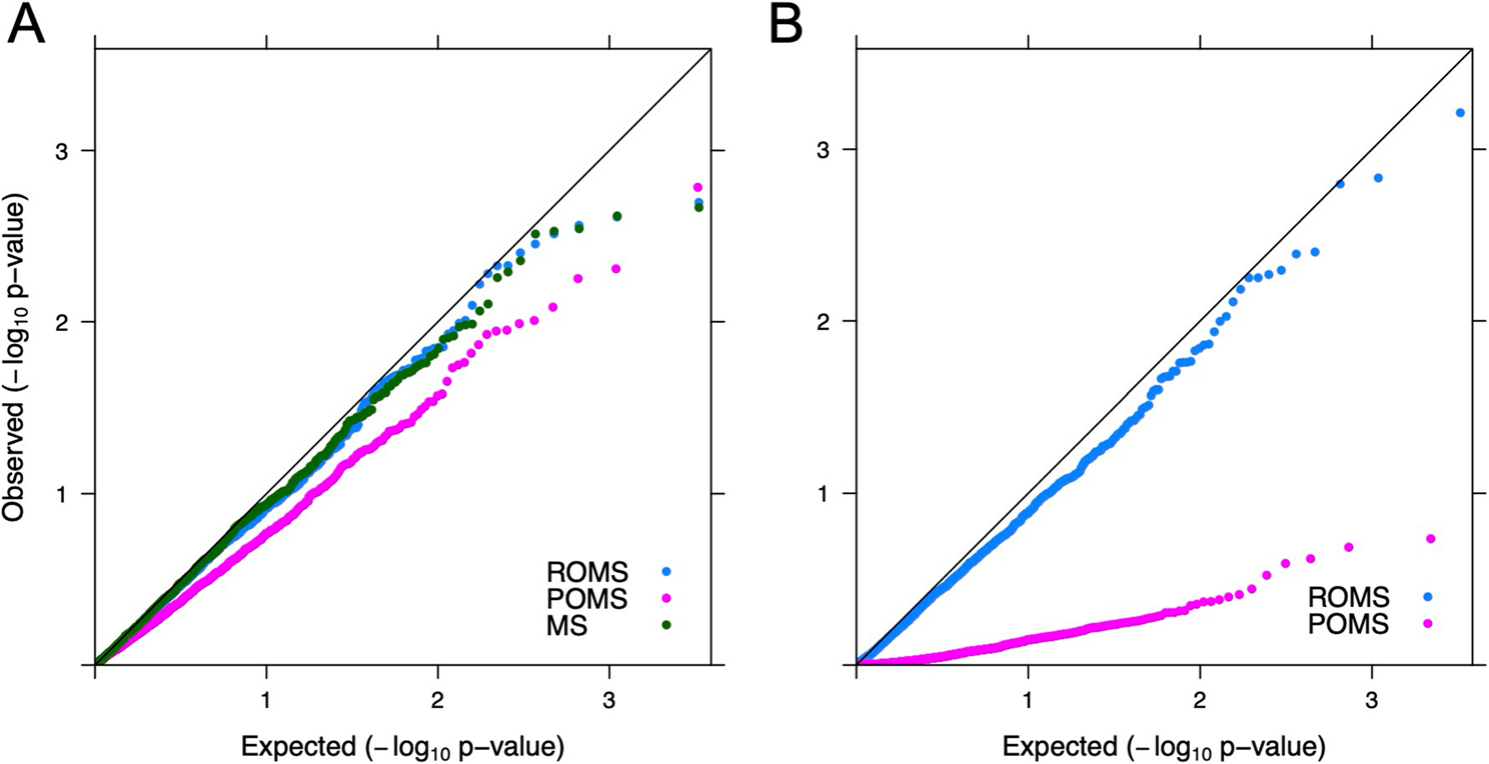
Quantile-quantile plots of gene burden association testing for progressive neurological disease genes. **A** Association testing by MS subtype; (**B**) Association testing with MS severity (ARMSS) MS: multiple sclerosis; POMS: progressing onset multiple sclerosis; ROMS: relapsing onset multiple sclerosis

To determine whether genetic variation in a larger number of progressive neurological disease genes is enriched in MS cases compared to controls, and therefore associated with MS risk, we investigated exome sequencing data for 1,680 genes identified in PanelApp Australia. Gene burden testing was performed using SKATO for variants that are both rare in European populations and predicted to be deleterious (CADD ≥ 15). Our analysis did not identify any genes with study-wide significant evidence of enrichment (applying an FDR adjusted *P*-value of < 0.05) when combining all MS cases, or when analysed separately by disease onset subtype. Overall, the Q-Q plot shown in Fig. 3A demonstrated a *P*-value distribution across all genes similar to that expected under the null hypothesis (Fig. 3A; Additional file 2: table S6).

An equivalent, cases only analysis, was also performed for MS severity using the age-related MS severity (ARMSS) score, analysed separately for ROMS and POMS. No gene demonstrated a study-wide significant association (FDR adjusted *P* < 0.05) of rare deleterious variation with ARMSS (Fig. 3B; Additional file 2: table S7) for either cases with POMS or ROMS.

## Discussion

MS diagnosis and onset of progressive neurological disability require consideration of other clinical explanations for the symptomology. Recently, Solomon and colleagues emphasised that clinicians must be conscious of ‘red flags’ during patient examination [33], or inconsistencies with the 2017 McDonald diagnostic criteria for MS, which may indicate the presence of an ‘MS mimic’. Such ‘red flags’ may include paraclinical findings such as brain MRI features consistent with an alternative diagnosis (e.g. pulvinar T1 hyperintensities consistent with Fabry disease), a positive family history of other neurological diseases, or acute emergence of new symptoms that are atypical for MS and may be non-neurological [33]. Several monogenic progressive neurological diseases could account for these ‘red flag’ observations.

Here, in a cohort of MS cases and controls recruited for genetic research, we have investigated the prevalence and impact of rare deleterious variation using exome sequencing. This has enabled a comprehensive examination of clinical variation in genes that cause monogenic forms of progressive neurological diseases in a large cohort of MS cases who received their diagnosis from sub-speciality clinics in Australia and New Zealand. Reassuringly we found that in this cohort the occurrence of MS mimics and genetic multimorbidity was rare, occurring at an approximate rate of 0.13%, affecting four individuals among the 3,071 MS cases for whom detailed clinical history review was possible. Only one of the four individuals was diagnosed with POMS. Our findings are comparable to results from a previous exome sequencing study of 278 MS cases that assessed variation in 495 genes, identifying one case with a disease-causing variant (0.36% of cohort) [15].

Previous research by Jia et al. [12] identified a significant enrichment of rare genetic variants in HSP disease genes in POMS in comparison to controls. In their study, this enrichment of variation in HSP genes in POMS was derived from 169 array-based variants and identified using a discovery sample of 48 array-genotyped POMS cases and 100 controls followed by replication in 266 POMS and 887 controls [12]. Given the important implications of their findings, that rare variants in Mendelian disease genes contribute to POMS development, we investigated the same 169 variants in our cohort to replicate their findings. Our results were discordant with the findings of Jia et al. as we found no evidence for enrichment of variation in HSP genes in POMS in our cohort. Several factors may have contributed to the discordance between our findings and those of Jia et al., including differences in discovery sample size, population background and approaches to case ascertainment [12]. The proposed role of Mendelian disease genes, such as HSP genes, in complex diseases like MS, has its basis in their overlapping biological pathways where different variant types in a gene may contribute to a spectrum of diseases. Given the biological and symptomology overlaps between MS and progressive neurological diseases we sought to assess whether rare, likely deleterious, genetic variants are associated with MS risk or severity at the gene level.

To determine whether other progressive neurological disease genes play a role in MS, we broadened the scope of the rare variant enrichment analyses of Jia et al. [12] to consider 1,680 genes reported by PanelApp Australia, which includes the established clinically important genes for HSPs. By drawing upon this broader unselected gene panel, we widened the scope of our analyses to consider a full spectrum of established neurological disease genes. Using gene-based testing with SKATO, we did not observe statistically significant enrichment of rare deleterious genetic variation in MS cases vs. controls, including either POMS or ROMS, or in disease severity extremes as measured by ARMSS. We note that the smaller sample size of the POMS group is reflected in a reduced statistical power for gene-based association testing in this group, leading to an under dispersion of *p*-value test statistics (Fig. 3). In light of these findings, we propose that there is a limited contribution for rare genetic variants in progressive neurological disease genes in MS overall. At the individual case level, however, we cannot rule out the possibility that rare variants contribute to the biological background upon which an individual’s MS has developed.

The four individuals identified in this study with pathogenic variants in clinically recognised genes demonstrate the potential for MS misdiagnosis and multimorbidity. We recognise that the cohort presented here is one that has been extensively reviewed by MS neurologists with sub-speciality expertise and is not necessarily representative of a generalist neurology clinic where a higher rate of MS misdiagnosis could occur. As genome and exome sequencing data becomes available in other studies, the implications of this finding warrant consideration in other established population-based MS cohorts. For example, individuals originally diagnosed with MS and included in a genetic study may later have their diagnosis changed, or in response to clinical history review may no longer satisfy the requirements of an MS diagnosis. The diagnostic criteria for MS continue to be refined [33], and while genetic testing for other diseases is not required, several of the diseases implicated by diagnostic ‘red flags’ are genetic, including spastic paraplegias (*KIF5A*) and CADASIL (*NOTCH3* – see also Mandler et al. [15]) as identified in this study, and can potentially be ruled in or out using clinical genome sequencing. Among the 75 MS cases for whom clinical history reviews were conducted, 71 did not exhibit evidence in their records of the likely pathogenic/pathogenic variant identified. Several factors may contribute to this, including incomplete penetrance of the variant, late-onset genetic disease, or a masking of the genetic disease by the individual’s MS. We note that our findings presented here are from data generated for research purposes, not from a clinical genomics pipeline, and we recognise that any return of results to participants in such a study requires additional accredited validation, variant curation, and genetic counselling.

## Conclusions

Our research suggests that for individuals diagnosed with MS by a neurologist with sub-speciality expertise, the rate of misdiagnosis, mimicry and/or multi-morbidity is very low. Nevertheless, in some rare instances, MS patients may benefit from clinical genetic testing for causal mutations in progressive neurological disease genes, particularly those with unusual paraclinical findings or ‘red flags’. Where an alternate genetic diagnosis is made, immediate benefits may include avoidance of side-effects from DMTs, reduced healthcare costs associated with DMTs and more appropriate management and genetic counselling.

## Supplementary Information

Additional file 1: Supplementary Material. File contains methods S1-S3.

Additional file 2: Supplementary Tables. File contains tables S1, S4-S7.

## Data Availability

Genomic data investigated in this study are the result of a research collaboration agreement (RCA) between The Florey Institute of Neuroscience and Mental Health and Regeneron Genetics Center^®^ (RGC™). The Florey entered into the RCA on behalf of the Australian and New Zealand MS Genetics Consortium (ANZgene), which is comprised of multiple institutions who contributed DNA samples and associated phenotype data for the RCA. RGC generated and own the genomic data but have provided the means for The Florey to license the genomic data for collaborative, academic research projects.ANZgene is a research platform of MS Australia and access to both genomic and phenotype data used in this study may be requested initially by contacting research@msaustralia.org.au. Requests for data access will be reviewed initially by the ANZgene Steering Committee to ensure the proposed research project aligns with institutional ethical approvals. A response to this initial request will take 2-3 weeks. For compliant projects, a controlled access, information transfer agreement (ITA) will be made available to principal investigators. The ITA is reflective of the terms and obligations contained in the RCA between Florey and Regeneron, which Florey is obliged to pass along to third party collaborators.It is envisaged that principal investigators will make available the ITA to their institutional legal representatives and they will work with Florey legal representatives toward execution of the ITA. It is anticipated that 4-6 weeks will be required for legal review and ITA execution, and data made accessible approximately 2 weeks thereafter.

## Abbreviations

ABMDR: Australian Bone Marrow Donor Registry
ANZgene: Australia and New Zealand MS Genetics Consortium
ARMSS: Age Related Multiple Sclerosis Severity Score
CADD: Combined Annotation-Dependent Depletion score
CADASIL: Cerebral autosomal dominant arteriopathy and subcortical infarct leukoencephalopathy
DMT: Disease-modifying therapy
EDSS: Expanded Disability Status Scale
FLAIR: Fluid-Attenuated Inversion Recovery
gDNA: Genomic DNA
GSA: Illumina Global Screening Array
HSP: Hereditary spastic paraplegia
MRI: Magnetic resonance imaging
MS: Multiple sclerosis
POMS: Progressive onset MS
ROMS: Relapsing onset MS
SNP: Single nucleotide polymorphism
SKATO: Sequence Kernal Association Test – Optimal
