# Supplementary material for: Rare variation in neurological disease genes and its role in multiple sclerosis mimicry and phenotype
Source: Genome Med. 2025 Dec 10;17:149. doi: 10.1186/s13073-025-01582-x (PMC12715922; doi:10.1186/s13073-025-01582-x)
Supplement: Supplementary file 1 — Additional file 1: Supplementary Material. File contains methods S1-S3. [file 13073_2025_1582_MOESM1_ESM.docx]

**Rare variation in neurological disease genes and its role in multiple sclerosis mimicry and phenotype**

Method S1: Whole exome sequencing, SNP array genotypes and quality control

1. Overview

The Australia and New Zealand MS Genetics Consortium (ANZgene) is an international collaboration and research platform of Multiple Sclerosis Australia (<https://www.msaustralia.org.au/anzgene>). In the current study, contributing ANZgene study sites were distributed across Australia (Melbourne (Victoria), Newcastle (New South Wales), Perth (Western Australia), Hobart (Tasmania)) and New Zealand. ANZgene sites independently recruited participants to their cohorts over a period of 25 years and contributed DNA samples and MS diagnosis and/or clinical histories to this study where available.

Samples were provided to the Regeneron Genetics Center (RGC) in two batches: an initial batch of 7,141 DNA samples (3,683 MS cases and 3,458 controls; Batch 1) and a second batch of 1,513 samples (1,459 MS cases and 54 controls; Batch 2). The Batch 1 samples were genotyped using the Infinium GSA SNP array platform, and whole-exome sequenced using IDT xGen custom exome capture followed by sequencing on an Illumina NovaSeq 6000 sequencer. Batch 2 samples were whole-exome sequenced using the Twist Comprehensive Exome Panel followed by sequencing on an Illumina NovaSeq 6000 sequencer. Fig S1 shows an overall flow chart of sample and genetic data quality control, as described in further detail in the full supplementary methods.

**Fig S1. Flow chart of sample and genetic data quality control**

GSA: Illumina Infinium Global Screening Array; IDT: Integrated DNA Technology; MS: multiple sclerosis; POMS: progressing onset multiple sclerosis; QC: quality control; RGC: Regeneron Genetics Center; ROMS: relapsing onset multiple sclerosis; WES: whole exome sequencing

2. Illumina GSA SNP genotyping

2.1 GSA genotyping and quality control undertaken by RGC

Illumina Infinium GSA genotyping of Batch 1 samples was conducted by genotyping 24 samples per array. The chip version was GSA-24v2-0_A2. A single sample failed multiple attempts at genotyping. RGC conducted in-house quality control on the 7,140 samples that successfully underwent array-based genotyping, and of these, 781 samples failed one or more of four in-house metrics including: genetic sex discordance with manifest-reported sex, genotype call rate less than 90%, array genotype data discordance with WES data or sample duplication. Exclusions are summarised in Table S2.

| **Table S2. RGC in-house GSA sample exclusions** | |
| --- | --- |
| **Metric failed** | **Count** |
| Sex discordant | 330 |
| Call rate < 90% | 607 |
| Exome discordant | 55 |
| Sample duplicated | 168 |
| Multiple metrics failed | 337 |
| Total samples failed | 781 |

After sample exclusions made by RGC, 6,359 GSA genotyped samples remained for further analysis. RGC provided genotype calls for this sample set only and we conducted our own in-house quality control on these samples, in addition to the steps already taken by RGC.

2.2 GSA quality control undertaken by ANZgene

Four samples with a neuromyelitis optica spectrum disorder (NMOSD) diagnosis were excluded, leaving 6,355 samples for analysis. Long range regions of high linkage disequilibrium (LD) were excluded as recommended by Price et al. (1) and Weale (2). An LD-pruned set of 397,437 variants was generated using PLINK v1.90 (3) (--indep-pairphase 20000 2000 0.5). Using 11,101 chromosome X variants, the X-chromosome inbreeding coefficient (F estimate) was calculated with PLINK v1.90 to confirm genetically determined sex against the manifest-reported sex. The maximum female F estimate threshold was set at 0.35, and the minimum male F estimate threshold was set at 0.4. Six samples were discordant with the manifest sex and were excluded from further analysis (Fig S2) leaving 6,349 samples for analysis.


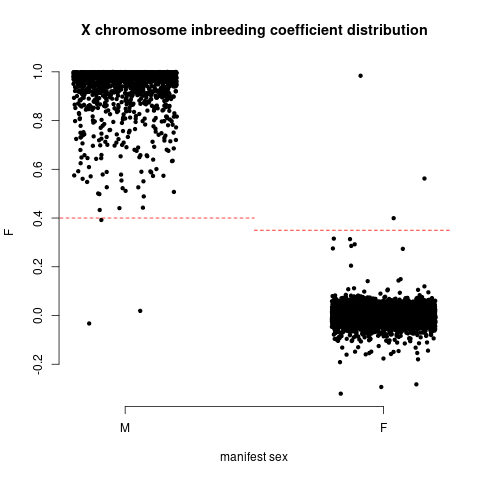


**Fig S2.** Calculation of sample sex within the GSA variant call set. Red dashed lines indicate the F estimate threshold used for each sex (M = male, F = female).

Per-sample heterozygosity and missingness were calculated using PLINK v1.90. Eight samples were identified as overt outliers of either metric (heterozygosity >8 standard deviations from the mean or >10% of genotypes missing) and were excluded (Fig S3), leaving 6,341 samples for analysis.


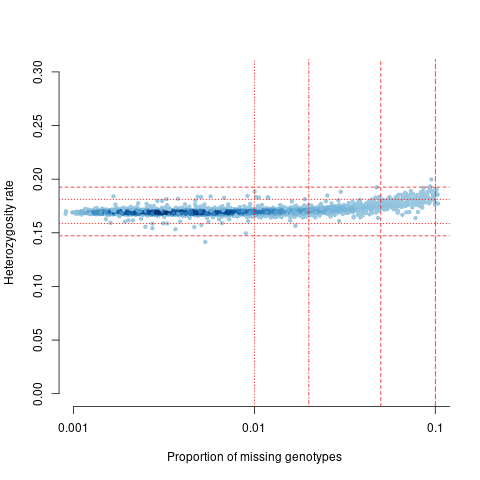


**Fig S3.** Calculation of heterozygosity and genotype missingness within the GSA variant call set. Red horizontal dotted lines indicate 4 standard deviations from the mean heterozygosity and red horizontal dashed lines indicate 8 standard deviations from the mean heterozygosity. Red vertical lines from left to right indicate a per sample genotype missingness of 1%, 2%, 5% and 10% respectively.

An analysis of relatedness using PRIMUS (4) within the 6,341 samples was conducted to identify first- through to third-degree relative pairs (Fig S4). In total, 68 first-degree, 26 second-degree, and 7,089 third-degree relative pairs were identified. The high number of third-degree relative pairs is driven by a smaller number of individuals inferred to have many relationships. One of these individuals was inferred to be related at third-degree level with 6,308 others, and a second was inferred to be related to 637 others, forming a large network that was resolved by the removal of these individuals. The next highest number of relationships participated in by a sample was 21. A maximum set of 6,171 unrelated individuals was generated using PRIMUS. In generating the maximum set of unrelated individuals, we prioritised the retention of MS cases.


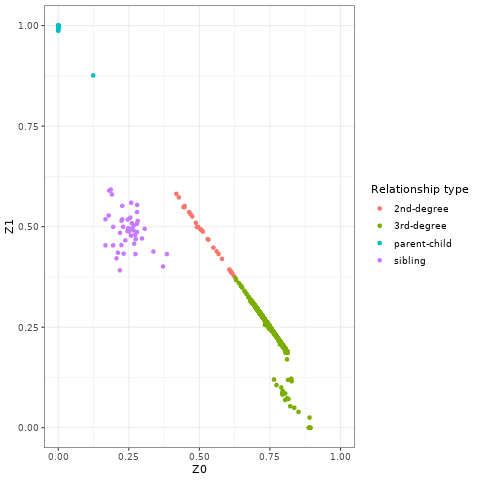


**Fig S4.** Pairwise relationships estimated for the 6,341 samples with GSA genotypes using PLINK v1.9. Unrelated samples are excluded.

2.3 Principal component analysis (PCA) to identify ancestry outliers for exclusion

Using the maximum unrelated sample of 6,171 individuals, PCA using SmartPCA version: 16000 (part of Eigensoft package v7.2.1) (5) was conducted to identify samples with outlying ancestry for exclusion from the sample set. Analysis was restricted to 374,359 autosomal biallelic SNPs with a call rate threshold of 0.95, MAF of 0.02 as per Belkadi et al. (6) and a Hardy–Weinberg equilibrium threshold of P = 10^-7^. Long range regions of high LD were excluded as recommended by Price et al. (1) and Weale (2).

The 6,171 ANZgene GSA samples were merged with the 1000 genomes project (1KGP) biallelic SNV reference panel (7). Merged variants with mismatched alleles were excluded. Variants with allele frequency < 0.01 in the 1KGP European super-population as well as variants with an allele frequency difference > 0.25 between the 1KGP European super-population and the ANZgene samples were excluded, after which 371,222 SNPs were retained. A final LD-pruning R^2^ threshold of 0.3 (using PLINK v1.90 with --indep-pairwise 1500 150 0.3) was used on the combined data set resulting in a set of 239,942 overlapping variants for analysis.

PCA was first conducted with 2,548 1KGP samples alone, with no outlier removal, and then the ANZgene samples were projected onto the 1KGP PC-space. Ancestry of the ANZgene samples, at the resolution of the five 1KGP super-populations (African, Admixed American, East Asian, European, and South Asian), was predicted as per the Peddy software package (8), which used the first 4 PCs from the 1KGP samples to train a support vector machine (SVM) for classification with a radial basis function kernel, implemented in R (9, 10) (Fig S5). Ancestry was predicted if the SVM prediction probability was > 0.65, otherwise sample ancestry was not predicted. The SVM parameters (kernel parameter, gamma and cost parameter (C)) were tuned by a 10-fold cross-validation on the 1KGP training data, identifying gamma = 8 and C = 2 parameter values. Within the ANZgene GSA data, a set of 6,055 samples were inferred to cluster with the 1KGP European super-population and these EUR-clustering samples were retained for further analysis.


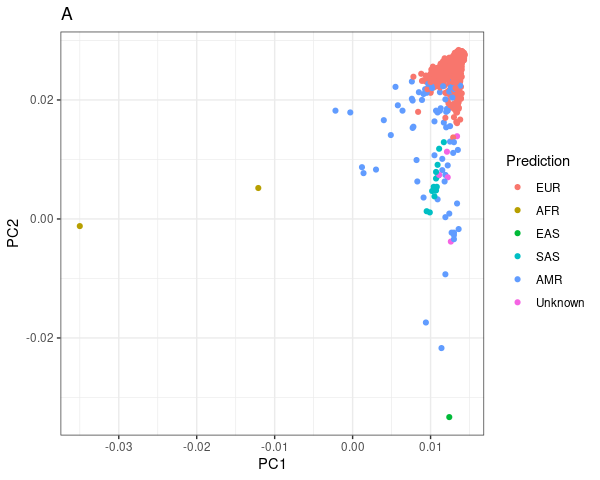

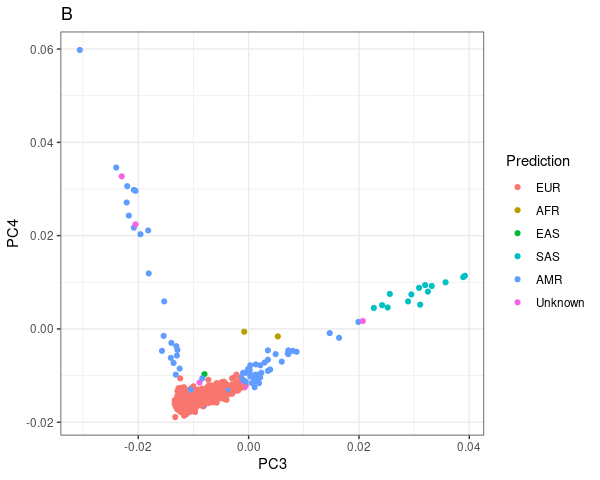


**Fig S5.** ANZgene GSA samples projected onto the top 4 PCs from 1KGP PCA. Samples are coloured by their predicted 1KGP super population.

2.4 GSA data final kinship check and PCA covariate calculation

Kinship calculations were rerun on the array data set of 6,055 EUR-clustering samples using the same variant set as the first round of kinship analysis. This confirmed that no individuals in the set were related at third-degree or closer. Then, within this set of 6,055 individuals, a PCA was conducted to identify PCs for inclusion as covariates in genetic analyses to control for latent population structure within the dataset. The set of SNPs used for PCA was obtained using identical QC thresholds as for the first PCA, however these being applied to the unrelated EUR-clustering samples resulted in 369,744 SNPs, which was reduced to 196,018 SNPs after LD-pruning. PCA was performed with no outlier removal and the top 20 PCs were obtained. Fig S6 and S7 may be used as a guide in deciding the number of PCs required for inclusion as covariates in genetic analyses to account for latent population structure.


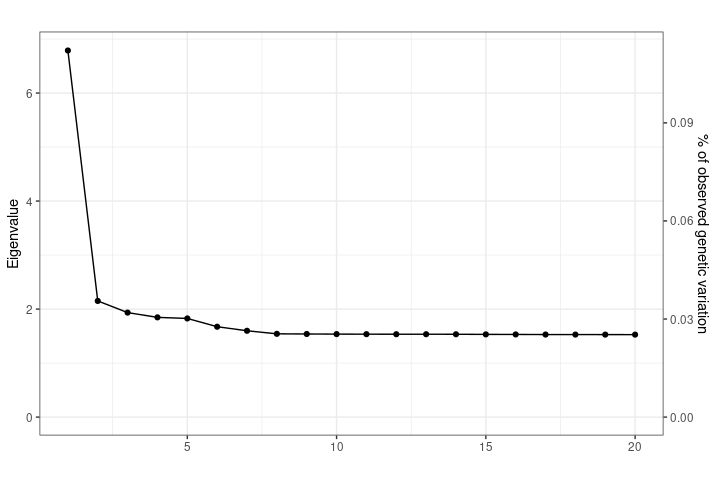


**Fig S6.** Eigenvalues from top 20 PCs in PCA with 6,045 unrelated EUR-clustering individuals with the array variant call set.


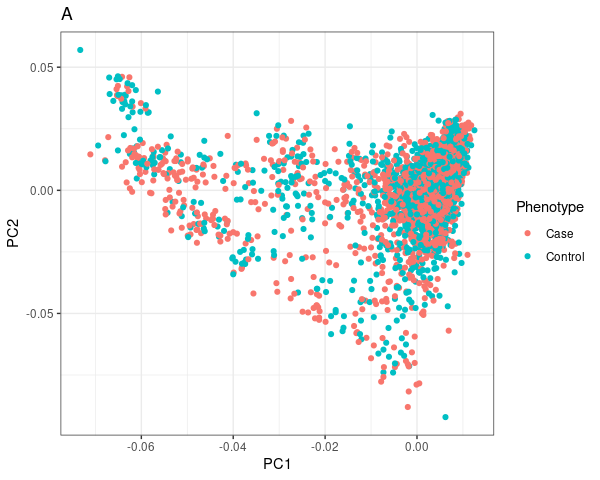

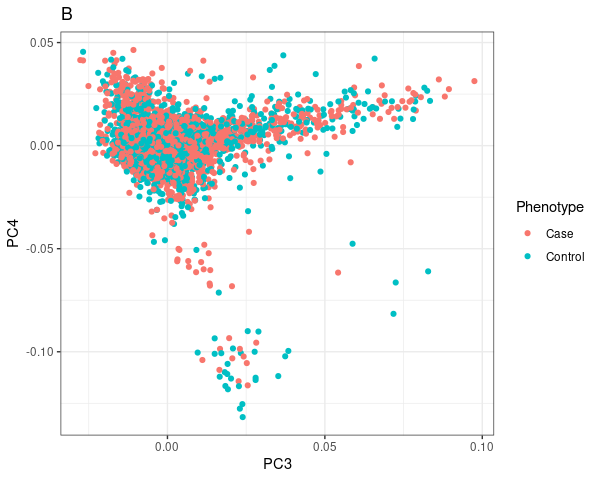


**Fig S7.** The top 4 PCs in PCA with 6,045 unrelated EUR-clustering individuals with the array variant call set.

3. Whole exome sequencing (WES)

3.1 WES and quality control undertaken by RGC

As described by Van Hout et al. (11), the RGC exome capture used for the Batch 1 samples was a modified version of the Integrated DNA Technologies (IDT) xGen Exome Research Panel v1.0. Of the 7,141 samples initially sent to RGC for WES, 352 samples had either insufficient DNA (N=107) or failed at the library preparation stage, after multiple attempts (N=245), leaving 6,789 samples successfully sequenced.

Samples from Batch 2 were prepared for sequencing using a custom automated sample preparation workflow developed at the RGC. Genomic DNA libraries were created by enzymatically shearing DNA to a mean fragment size of 200 base pairs using reagents from New England Biolabs. A common Y-shaped adapter (IDT) was ligated to all DNA libraries. Unique, asymmetric 10 base pair barcodes were added to the DNA fragments during library amplification with Kapa HiFi to facilitate multiplexed exome capture and sequencing. Equal amounts of sample were pooled prior to overnight exome/genotype capture with the Twist Comprehensive Exome panel, RGC developed Twist Diversity SNP panel, and additional spike-ins to boost coverage at selected CHIP sites and to cover the mitochondrial genome; all samples were captured on the same lot of oligos. The captured DNA was PCR amplified and quantified by qPCR. The multiplexed samples were pooled and then sequenced using 75 bp paired-end reads with two 10 bp index reads on the Illumina NovaSeq 6000 platform on S4 flow cells. Of the 1,513 samples sent to RGC, 33 samples failed at the library preparation stage after multiple attempts, and three duplicate samples were not processed, leaving 1,477 samples successfully sequenced.

For the 8,266 samples that were successfully sequenced across both batches, following the OQFE (original quality functional equivalent) protocol (12), sequence reads were mapped to GRCh38 references using BWA MEM (13) in an alt-aware manner, read duplicates were marked, and additional per-read tags were added. Single nucleotide variants (SNVs) and short insertions and deletions (indels) were identified using a Parabricks accelerated version of DeepVariant v0.10 with a custom WES model and reported in per-sample genome VCF (gVCF) files. These exome gVCFs were aggregated with GLnexus v1.4.3 using the pre-configured DeepVariantWES setting (14) into a joint-genotyped multi-sample project-level VCF (pVCF), which was converted to bed/bim/fam format using PLINK v1.90. (3).

RGC excluded 301 samples for failing one or more of five in-house metrics including: genetic sex discordance with manifest-reported sex, 80% of capture covered at < 20X, contamination >5% detected, duplication of samples sent (within each batch), or WES-derived genotype discordance with genotyping data. Exclusions are summarised in Table S3.

| **Table S3. RGC in-house WES sample exclusions** | |
| --- | --- |
| **Metric failed** | **Count** |
| Sex discordant | 59 |
| 20X < 80% | 34 |
| Contamination > 5% | 55 |
| Sample duplicated | 186 |
| Genotype discordant | 26 |
| Multiple metrics failed | 59 |
| Total | 301 |

After RGC exclusions, 7,965 WES samples remained for further analysis and RGC provided a joint variant call set for these samples only. We conducted our own in-house quality control on WES data from these samples, in addition to the steps taken by RGC.

3.2 ANZgene quality control conducted on WES DeepVariant call set

Eight samples with neurological diagnoses other than MS were excluded, leaving 7,957 samples for analysis. Long-range regions of high LD were excluded as recommended by Price et al. (1) and Weale (2). An LD-pruned set of 1,585,408 variants was generated using PLINK v1.90 (3) (--indep-pairphase 20000 2000 0.5). Using 37,793 chromosome X variants, the X-chromosome inbreeding coefficient (F estimate) was calculated with PLINK v1.90 to confirm genetically determined sex against the manifest-reported sex. The maximum female F estimate threshold was set at 0.5, and the minimum male F estimate threshold was set at 0.6. Four samples were discordant with the manifest-reported sex and were excluded from further analysis (Fig S8), leaving 7,953 samples.


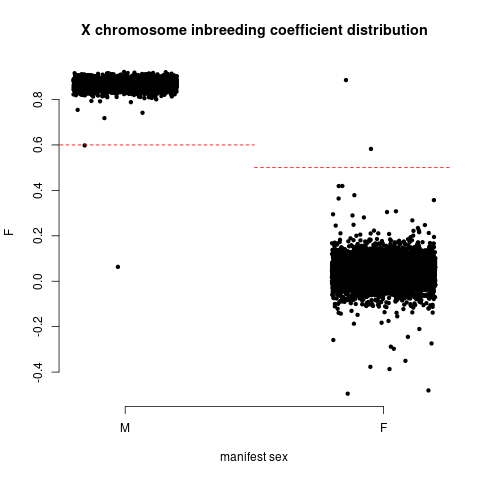


**Fig S8.** Calculation of sample sex within the WES GL variant call set. Red dashed lines indicate the F estimate threshold used for each sex (M = male, F = female).

Per sample heterozygosity and missingness were calculated using PLINK v1.90. Three samples were identified as heterozygosity outliers and excluded (Fig S9), leaving 7,950 samples for analysis.


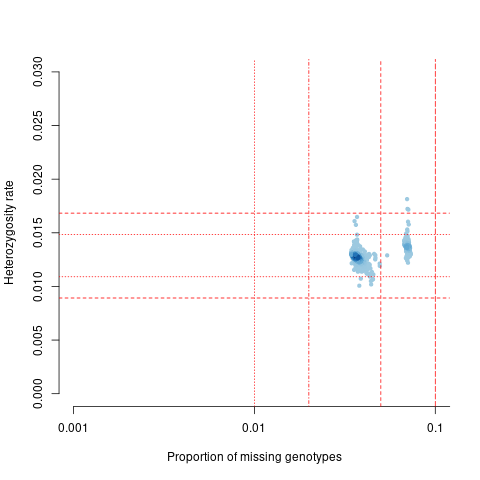


**Fig S9.** Calculation of heterozygosity and genotype missingness within the WES variant call set. Red horizontal dotted lines indicate 4 standard deviations from the mean heterozygosity and red horizontal dashed lines indicate 8 standard deviations from the mean heterozygosity. Red vertical lines from left to right indicate a per sample genotype missingness of 1%, 2%, 5%, and 10% respectively. Note, all samples had less than 10% of genotypes missing.

An analysis of relatedness using PRIMUS (4) within the 7,950 samples was conducted to identify first through to third degree relative pairs (Fig S10). In total, 101 first degree, 76 second degree, and 257 third degree relative pairs were identified, as well as 143 duplicate samples that were sequenced in both batches. A maximum set of 7,590 unrelated individuals was generated using PRIMUS. In generating the maximum set of unrelated individuals, we first prioritised the retention of MS cases, then Batch 1 samples, then samples with lower missing genotype rates.


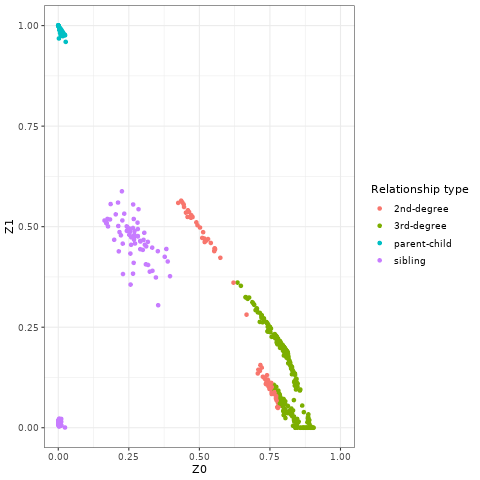


**Fig S10.** Pairwise relationships estimated for the 7,950 exome sequenced samples, using PLINK v1.90. Unrelated samples are excluded.

3.3 Principal component analysis (PCA) to identify ancestry outliers for exclusion

An identical PCA and ancestry prediction was applied as for the GSA data, starting with 121,072 autosomal biallelic SNPs passing initial QC, which was reduced to 52,392 SNPs after merging with 1KGP data, and finally 239,942 SNPs after LD-pruning. Within the ANZgene WES data, a set of 7,203 samples were inferred to cluster with the 1KGP European super-population (Fig S11) and these EUR-clustering samples were retained for further analysis.


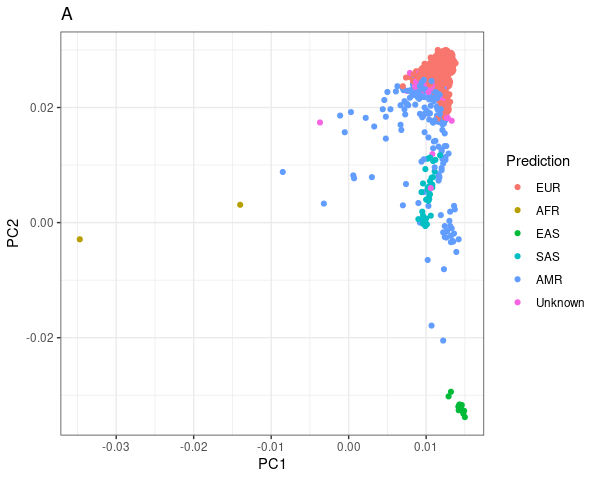

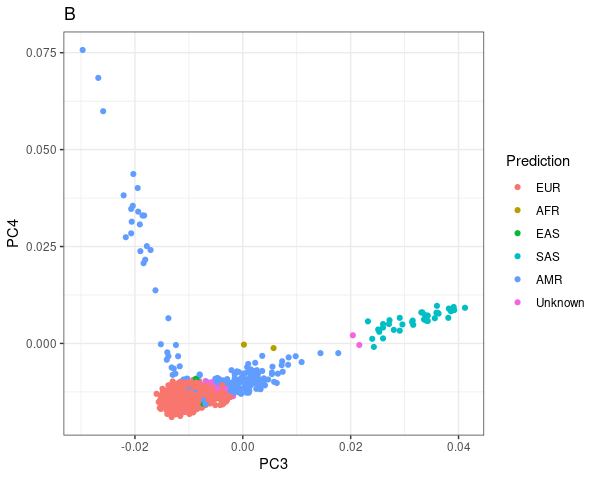


**Fig S11.** ANZgene WES samples projected onto the top 4 PCs from 1KGP PCA. Samples are coloured by their predicted 1KGP super population.

3.4. A saliva- vs blood-derived DNA comparison of variant calls from WES data

Of the 7,141 Batch 1 DNA samples provided to RGC, 5,763 samples were derived from whole blood and 1,378 were derived from saliva. All saliva-derived DNA samples came from MS cases, the majority originating from New Zealand (n=1,366) and 12 from Tasmania, Australia (15). On provision of WES data to ANZgene, RGC made it known that some variant calls stratified between saliva- and blood-derived DNA samples, suggesting the potential for a technically induced artefact. To determine the impacted variants/genomic regions, the ANZgene data analysis team conducted a dedicated investigation to identify and exclude any variants that may be subject to this type of technical artefact.

Within the maximally unrelated set of 7,203 EUR-clustering samples, 3,253 were MS cases from Batch 1. A genome-wide analysis was conducted comparing variant frequencies in samples sequenced from saliva-derived DNA (N=1,277) vs samples sequenced from blood-derived DNA (N=1,976). To reconcile the cause of the saliva–blood variant stratification, we hypothesised that next-generation sequencing of particular regions of the genome may be affected by alignment of non-human sequences within saliva samples, which may in turn affect variant calling. We therefore aimed to identify these regions in the WES data and exclude variants within the equivalent of one read length (±75 bp) either side of associated variants from the analysis.

Our analysis controlled for the first four PCs, to account for potential bias in sample origin, with all DNA samples from New Zealand and eight from Tasmania being saliva-derived, while all remaining samples (from multiple locations across Australia) were blood-derived. Based on the distribution of the association result p-values (Fig S12) we set a conservative threshold of P = 0.00025 to define regions for exclusion, excluding all variants within 75bp upstream and downstream of variants with P < 0.00025. There were 100 variants associated at this significance level (Fig S13), which co-located with 88 genomic regions.


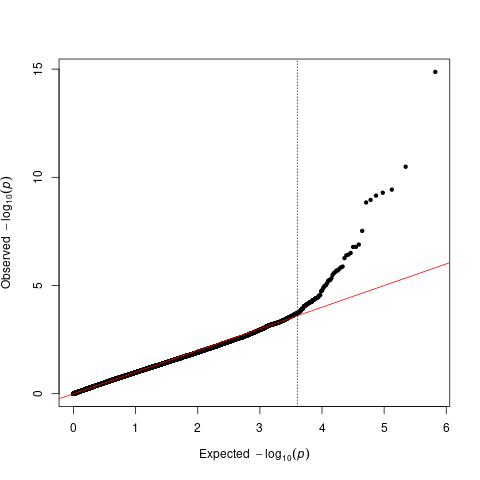


**Fig S12.** Quantile–quantile plot of the saliva-derived DNA vs blood-derived DNA genome-wide associations. The red dashed line shows the conservative threshold of P = 0.00025 used to identify associated variants/regions for exclusion.


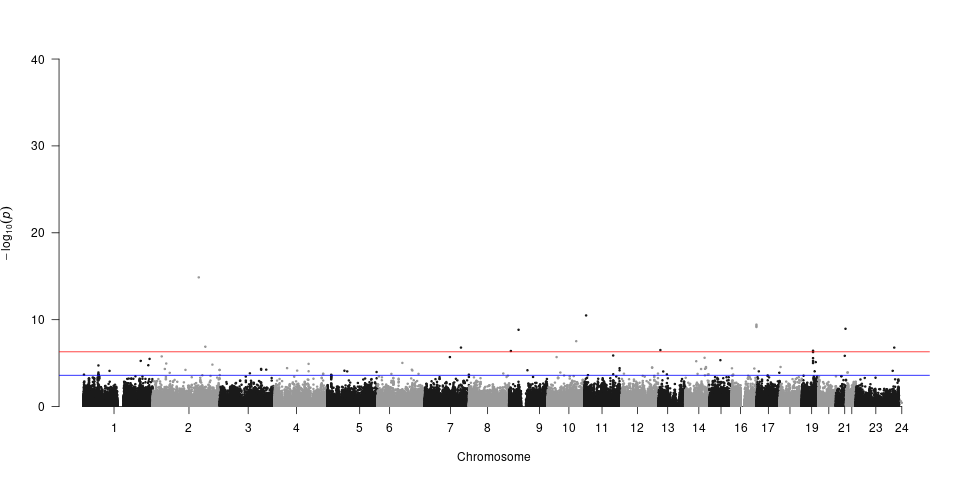


**Fig S13.** Manhattan plot showing results of the saliva-derived DNA vs blood-derived DNA genome-wide association analysis for the WES data. The blue line shows the conservative threshold of P = 0.00025 used to identify associated variants/regions for exclusion, and the red line shows the standard exome-wide significance threshold of P = 5 × 10^-7^.

3.5 WES data final kinship check and PCA covariate calculation

Kinship calculations were re-run on the array data set of 7,203 EUR-clustering samples using the same variant set as the first round of kinship analysis, after excluding variants in the 88 genomic regions identified from the saliva/blood comparison. This resulted in the removal of two individuals identified as having third-degree relationships. PCA was then conducted to identify PCs for inclusion as covariates in genetic analyses to control for latent population structure within the dataset. The set of SNPs used for PCA was obtained using identical QC thresholds as for the first PCA, however, these being applied to the unrelated European samples resulted in 121,072 SNPs, which was reduced to 45,870 SNPs after the removal of variants in saliva-associated regions and LD-pruning. PCA was performed with no outlier removal and the top 20 PCs were obtained. Fig S14 and S15 may be used as a guide in deciding the number of PCs required for inclusion as covariates in genetic analyses to account for latent population structure.


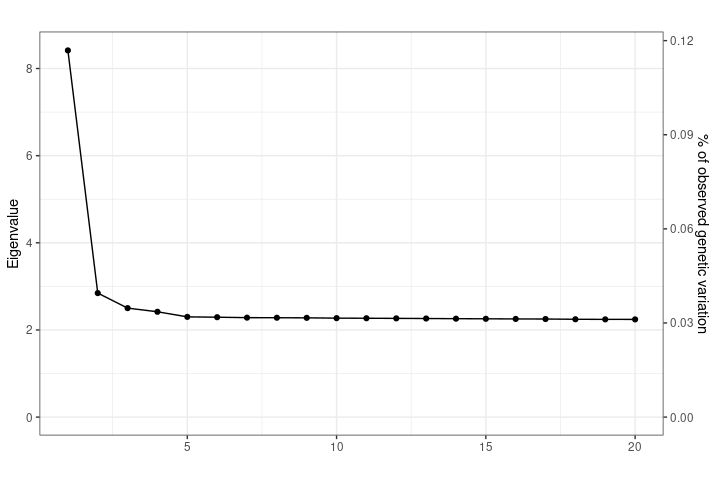


**Fig S14.** Eigenvalues from top 20 PCs in PCA with 7,201 unrelated EUR-clustering individuals with the exome variant call set.


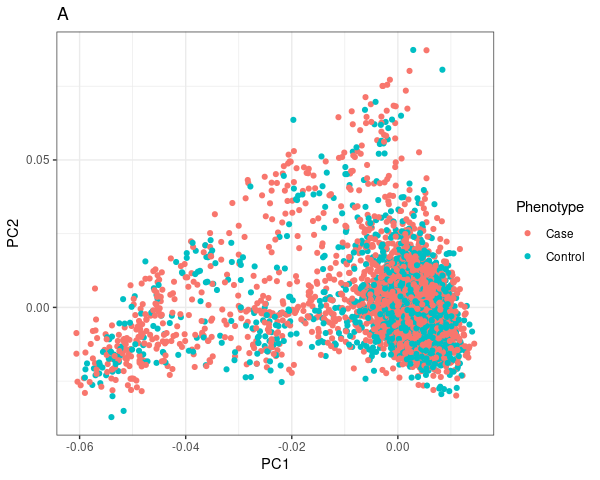

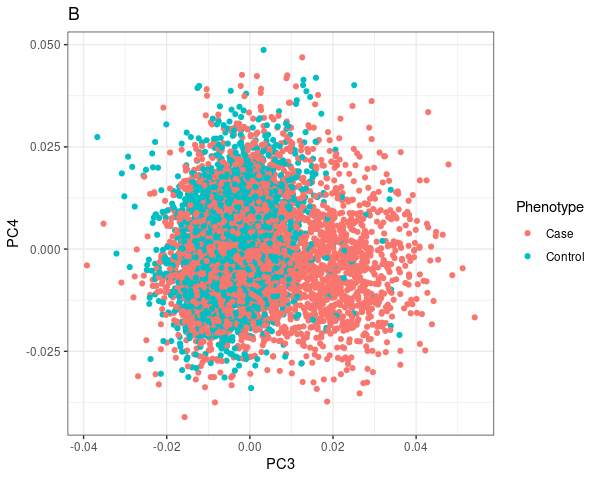


**Fig S15.** The top 4 PCs in PCA with 7,201 unrelated EUR-clustering individuals with the exome variant call set.

4. Final data processing

Variants in the 88 genomic regions identified from the saliva/blood comparison were removed from Batch 1 samples only. For both the GSA and WES variant sets, from a maximally unrelated sample of EUR-clustering individuals, a final variant filter was applied to remove variants with no calls or minor allele frequency of 0% across the dataset. These uninformative variants had been identified in early stages of data QC and retained after progressive filtering of samples. Ten samples with >10% of genotypes missing after the final variant filter were removed from the GSA dataset.

The final WES dataset consisted of 2,793,584 variants in 7,201 individuals (n=4,340 MS cases and n=2,891 controls) and the final GSA dataset consisted of 586,269 variants in 6,045 individuals (n=3,307 MS cases and n=2,738 controls).

Method S2: Identifying putative likely pathogenic and pathogenic variants in progressive neurological disease genes in MS cases

To identify variants in established progressive neurological disease genes, the exome variant set generated was converted using PLINK (v2.00a2.3LM) to VCF file format. Then BCFtools (v1.15.1) (16) was used to filter the VCF to the final set of exome sequenced individuals (Figure 1). For each variant, minor allele counts were calculated in PLINK to subsequently remove, using BCFtools, any variant now not occurring in the dataset because of sample filtering. Variants were annotated using Ensembl’s Variant Effect Predictor (VEP, v110) (17) outputting all possible variant-transcript combinations. Variants annotated to genes in the PanelApp Australia (18, 19) Progressive Neurological Diseases Superpanel ‘Green’ tier gene list (v14.477) were extracted. Analysis was restricted to 1,680 nuclear genes. Mitochondrial genes (N=35) were excluded as the mitochondrial genome was not captured in the exome sequencing designs. Nuclear genes (N=26) recorded in the PanelApp gene list as disease causing due to short tandem repeat expansions were also excluded.

Annotations from the ClinVar database release v20220910 were initially used to identify variants recorded as having a clinical significance of ‘Likely Pathogenic’ and/or ‘Pathogenic’. As analyses progressed across the duration of the study ClinVar annotations were updated to the v20240107 release with results presented with respect to this release of ClinVar. Variants that were identified from the v20220910 database that later had a change in clinical significance classification in the v20240107 database were still considered and are noted accordingly. Variants that had a ‘conflicting interpretation of pathogenicity’ designation in ClinVar, were included only if they met the criteria of 10 or more ClinVar entries with at least 50% of the entry classifications being ‘Likely Pathogenic’ or ‘Pathogenic’. Genes were then split based on their recognised mode of inheritance described by PanelApp Australia. Genes with a ‘biallelic, autosomal or pseudoautosomal’ or an ‘unknown’ mode of inheritance were investigated under a recessive model. This required carriers to be homozygous for a variant, or to have two variants in the same gene as a potential compound heterozygous carrier. Genes with inheritance models corresponding to ‘monoallelic’, or ‘X-linked’ inheritance were investigated under a dominant model of inheritance. This required carriers to be at least heterozygous for the identified variant. Due to there being multiple recognised inheritance patterns for many disease genes some genes were considered under both models of inheritance and resolved at the individual variant level.

Analysis was then restricted to variant identification in MS cases for whom access to clinical histories was achievable. The corresponding literature-based evidence for the variants meeting the criteria for this analysis was then assessed, variant by variant. Variants that were reported in ClinVar for a non-neurological disease (eg. cancer) were excluded from further analysis. The established model of disease inheritance from the literature, as well as supporting publications, were also reviewed for each variant to generate a prioritised list of cases for clinical follow-up.

For each variant and variant carrier, sequencing evidence was reviewed manually in the exome CRAM files using the Integrative Genomics Viewer (IGV, v2.11.9) (20) as a secondary confirmation of the genotype calls prior to clinical history reviews.

Method S3: Gene-based burden testing of progressive neurological disease genes in cases and controls

To mitigate potential confounding from exome capture effects, this analysis was restricted to MS cases and controls that were sequenced on the IDT exome capture, and those that had corresponding GSA array-based genotypes (Fig S1). Array based genotypes were used to fit whole genome regression models for each phenotype tested, and as the source of principal component covariates. The 388,245 variants identified in progressive neurological disease genes were restricted to 76,942 variants that passed a Hardy-Weinberg equilibrium exact test (P < 1 × 10^−15^), had a minor allele frequency ≤ 1% in the gnomAD non-Finnish European population, using allele frequencies from both gnomAD genome and exome datasets (v4.1), and a CADD (v1.6) Phred score ≥ 15 to identify rare variants with an *in silico* prediction of deleteriousness. Of the 1,680 PanelApp progressive neurological disease genes, 1,678 had qualifying variants. Gene-based testing was performed using the Sequence Kernel Association Test – Optimal (SKATO) (21), implemented in regenie (v3.4.1) and implementing the Cauchy combination method modification (SKATO-ACAT) for combining p-values from SKATO models (22). Variants were aggregated by gene into a mask for SKATO association testing. A Bonferroni significance threshold of 2.97 × 10^-5^ (0.05/1678) was used for each analysis. Analyses conducted in regenie were controlled for sex, and 10 principal components to control for population structure. All cases were analysed together as one MS group, and separately as a ROMS group, and a POMS group.

To test for association with MS disability and disease progression, the Age Related Multiple Sclerosis Severity Score (ARMSS) was calculated as per Manouchehrinia et al. (23), and analysed separately for ROMS and POMS individuals. The ARMSS distributions were tested for normality using the Shapiro-Wilk normality test in R (v4.3.1), and as both distributions were not normal (ROMS p-value < 2.2×10^−16^, POMS p-value < 2.1×10^−10^), rank inverse normal transformations were applied to the calculated ARMSS quantitative phenotypes. Analyses were controlled for sex, and 10 principal components to control for population structure.

References

1. Price AL, Weale ME, Patterson N, Myers SR, Need AC, Shianna KV, et al. Long-range LD can confound genome scans in admixed populations. American Journal of Human Genetics. 2008;83(1):132-5.

2. Weale ME. Quality control for genome-wide association studies. Methods Mol Biol. 2010;628:341-72.

3. Chang CC, Chow CC, Tellier LCAM, Vattikuti S, Purcell SM, Lee JJ. Second-generation PLINK: rising to the challenge of larger and richer datasets. GigaScience. 2015;4(1):7-.

4. Staples J, Nickerson DA, Below JE. Utilizing graph theory to select the largest set of unrelated individuals for genetic analysis. Genetic Epidemiology. 2013;37(2):136-41.

5. Patterson N, Price AL, Reich D. Population structure and eigenanalysis. PLoS Genet. 2006;2(12):190-.

6. Belkadi A, Pedergnana V, Cobat A, Itan Y, Vincent QB, Abhyankar A, et al. Whole-exome sequencing to analyze population structure, parental inbreeding, and familial linkage. Proceedings of the National Academy of Sciences of the United States of America. 2016;113(24):6713-8.

7. The 1000 Genomes Project Consortium. A global reference for human genetic variation. Nature. 2015;526(7571):68-74.

8. Pedersen BS, Quinlan AR. Who's who? Detecting and resolving sample anomalies in human DNA sequencing studies with peddy. American Journal of Human Genetics. 2017;100(3):406-13.

9. R Development Core Team. R: A language and environment for statistical computing. Vienna, Austria: R Foundation for Statistical Computing; 2011.

10. Meyer D, Dimitriadou E, Hornik K, Weingessel A, Leisch F. e1071: Misc Functions of the Department of Statistics, Probability Theory Group (Formerly: E1071), TU Wien. 2021.

11. Van Hout CV, Tachmazidou I, Backman JD, Hoffman JD, Liu D, Pandey AK, et al. Exome sequencing and characterization of 49,960 individuals in the UK Biobank. Nature. 2020;586(7831):749-56.

12. Krasheninina O, Hwang Y-C, Bai X, Zalcman A, Maxwell E, Reid JG, et al. Open-source mapping and variant calling for large-scale NGS data from original base-quality scores. bioRxiv. 2020:2020.12.15.356360-2020.12.15.

13. Li H, Durbin R. Fast and accurate short read alignment with Burrows-Wheeler transform. Bioinformatics. 2009;25(14):1754-60.

14. Lin MF, Rodeh O, Penn J, Bai X, Reid JG, Krasheninina O, et al. GLnexus: joint variant calling for large cohort sequencing. bioRxiv. 2018:343970-.

15. The Australia and New Zealand Multiple Sclerosis Genetics Consortium (ANZgene). Genome-wide association study identifies new multiple sclerosis susceptibility loci on chromosomes 12 and 20. Nature Genetics. 2009;41(7):824-8.

16. Danecek P, Bonfield JK, Liddle J, Marshall J, Ohan V, Pollard MO, et al. Twelve years of SAMtools and BCFtools. Gigascience. 2021;10(2).

17. McLaren W, Gil L, Hunt SE, Riat HS, Ritchie GR, Thormann A, et al. The Ensembl Variant Effect Predictor. Genome Biol. 2016;17(1):122.

18. Martin AR, Williams E, Foulger RE, Leigh S, Daugherty LC, Niblock O, et al. PanelApp crowdsources expert knowledge to establish consensus diagnostic gene panels. Nat Genet. 2019;51(11):1560-5.

19. Stark Z, Foulger RE, Williams E, Thompson BA, Patel C, Lunke S, et al. Scaling national and international improvement in virtual gene panel curation via a collaborative approach to discordance resolution. Am J Hum Genet. 2021;108(9):1551-7.

20. Robinson JT, Thorvaldsdottir H, Wenger AM, Zehir A, Mesirov JP. Variant Review with the Integrative Genomics Viewer. Cancer Res. 2017;77(21):e31-e4.

21. Lee S, Emond MJ, Bamshad MJ, Barnes KC, Rieder MJ, Nickerson DA, et al. Optimal unified approach for rare-variant association testing with application to small-sample case-control whole-exome sequencing studies. Am J Hum Genet. 2012;91(2):224-37.

22. Mbatchou J, Barnard L, Backman J, Marcketta A, Kosmicki JA, Ziyatdinov A, et al. Computationally efficient whole-genome regression for quantitative and binary traits. Nat Genet. 2021;53(7):1097-103.

23. Manouchehrinia A, Westerlind H, Kingwell E, Zhu F, Carruthers R, Ramanujam R, et al. Age Related Multiple Sclerosis Severity Score: Disability ranked by age. Mult Scler. 2017;23(14):1938-46.
